# Cataloguing guanidinoacetic acid content in nutritional supplements

**DOI:** 10.1002/fsn3.3206

**Published:** 2022-12-23

**Authors:** Sergej M. Ostojic, Laszlo Ratgeber, Nenad Trunic, Sava Rajkovic

**Affiliations:** ^1^ Applied Bioenergetics Lab, Faculty of Sport and Physical Education University of Novi Sad Novi Sad Serbia; ^2^ Department of Nutrition and Public Health University of Agder Kristiansand Norway; ^3^ Faculty of Health Sciences University of Pécs Pécs Hungary; ^4^ National Basketball Academy Pécs Hungary

**Keywords:** creatine, DSLD, glycocyamine, nutritional supplement

## Abstract

Guanidinoacetic acid (GAA, also known as glycocyamine or guanidinoacetate) is a naturally occurring alpha amino acid derivative and newly recognized dietary compound obtainable by different foods and nutritional supplements. Anecdotal evidence suggests that the GAA exposure from supplements might be a major source of GAA supply, out‐competing other food sources for several orders of magnitude. This original technical paper summarizes information about GAA levels in different nutritional supplements, as derived from the U.S. National Institutes of Health dietary supplement database.

## Graphical abstract

The amount of guanidinoacetic acid (GAA) in nutritional supplements registered in the U.S. National Institutes of Health dietary supplement database varies considerably. GAA‐containing products appear to be a significant source of GAA in human nutrition; further studies are needed to assess the risks and benefits of GAA exposure from nutritional supplements.

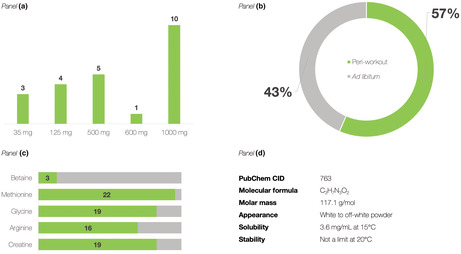

## 1 INTRODUCTION

Guanidinoacetic acid (GAA, also known as glycocyamine) is a naturally occurring alpha amino acid derivative and newly recognized dietary compound. GAA plays several essential roles in the human body (Ostojic, 2015), predominantly acting as a direct precursor of creatine, a critical molecular facilitator of cellular bioenergetics (Wallimann et al., 2011). GAA is synthesized endogenously from non‐essential amino acids glycine and *L*‐arginine in the human kidney and pancreas, but could also be obtained from a regular diet. Recently, the quantity of GAA in different foods has been categorized, with GAA identified in meat‐based foods, milk, and several plants (Ostojic, 2022). Besides a regular diet, exogenous GAA might also be consumed as a nutritional supplement, with GAA often recognized as a popular component of various supplemental mixtures aimed at improving exercise performance (Deldicque & Francaux, 2016). However, how much GAA is available from this source remains currently unknown, with anecdotal evidence suggesting that GAA from nutritional supplements might be a major source of GAA supply. This technical paper outlines information about GAA levels in different nutritional supplements, as derived from the U.S. National Institutes of Health (NIH) dietary supplement database.

## 2 METHODS

The NIH Office of Dietary Supplements manages the Dietary Supplement Label Database (DSLD), a publicly available open‐access data set containing all information printed on labels of dietary supplement products sold in the United States from 2011 onwards. Besides other details, DSLD contains information about the amount of dietary ingredients for over 143,000 nutritional supplements (National Institutes of Health, 2022). For this report, we searched DSLD using the following synonyms for GAA: guanidinoacetic acid, guanidinoacetate, glycocyamine, and betacyamine. The search revealed a total of 118 dietary supplements containing GAA, with the three most recent products added to the database in March 2021. Of the total number of GAA‐containing dietary supplements, 82 products were currently available in the U.S. market, and those products were analyzed further.

## 3 RESULTS

**FIGURE 1:**
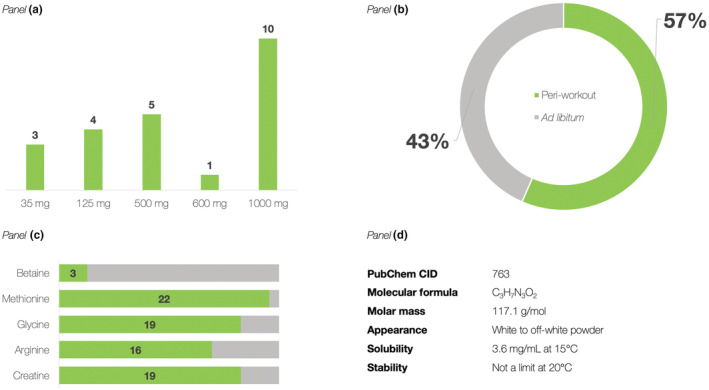
The summary of products containing guanidinoacetic acid (GAA) identified in the NIH dietary supplement label database, depicting the number of products with specified amount of GAA per serving (Panel a), the main domains of suggested use as extracted from the individual labels (Panel b), and the five most common dietary compounds mixed with GAA found in a supplement complex (number of products, of 23) (Panel c); (Panel d) details fundamental chemical properties of GAA.

Only 19 products (23.2%) were manufactured by a parent U.S. company, while other products companies were registered in DSLD as non‐manufacturers (e.g., distributor, packager, reseller, other), which makes it challenging to track down the origin of GAA in those products. The products containing GAA were typically in the form of powder (66 of 82 products, 80.5%), followed by caplets (13 products, 15.9%), and liquid supplement forms (3 products, 3.7%). A majority of GAA supplements labeled their use before, during, or after exercise (50 of 82 products, 61.0%), while 24 products (29.3%) suggested consumption during regular meals; the remaining eight products (9.7%) provided no recommendation for suggested use at their labels. Unfortunately, most entries (59 products, 72.0%) provided no information about the exact GAA content in a particular supplement and enlisted GAA only as a component of various proprietary blends. These products typically combined GAA with creatine and similar amino acids, with the total amount per serving for proprietary blends varying highly from 72.5 mg to 38.0 g. A total of 23 products (28.0%) labeled the exact amount of GAA per single serving, which ranged from 35 mg (three products) to 1000 mg (10 products) (Figure 1). All supplements in this subgroup were combination products, provided in powder form only; no single product indexed in DSLD contained GAA alone. More than half supplements (13 products, 56.5%) were directed to be consumed as a component of peri‐workout supplementation. The intended target group(s) included human consumers, 4 years and above, as classified within the database.

## 4 DISCUSSION

Nutritional supplements appear to be a relevant source of GAA, representing a wide range of GAA content where high‐potency products could supply ~30 times more GAA per single serving than low‐ranking parallels. In addition, the high‐level products could provide a substantially higher dietary exposure to GAA as compared to regular foods. For instance, pork and poultry render up to 160 mg of GAA per kilogram of food (Ostojic, 2022), while GAA supplements can provide up to 1000 mg per single serving. The average intake of GAA from a regular diet in a general population appears to be ~10 mg per day (Ostojic et al., 2022), while supplemental GAA (if consumed) could provide up to two orders of magnitude more GAA than a diet. This put forward nutritional supplements as highly concentrated sources of GAA, which should account for a total GAA turnover in future nutritional and metabolic studies with this compound. No dietary requirements for GAA have been established yet. Therefore, the biomedical relevance of being exposed to GAA from dietary supplements remains elusive. This is particularly important for the safety of high‐potency GAA supplements (Deldicque & Francaux, 2016) which requires well‐sized long‐term pharmacovigilance studies to address this issue. Besides, more efficacy studies are warranted to investigate the effects of daily intake of high‐dose GAA supplements versus low‐dose GAA supplementation and perhaps set up an effective and safe threshold for GAA intake. The fact that most GAA‐containing products indexed in DSLD presented no information about GAA content raises additional concerns about dietary exposure to GAA from these supplements.

## 5 CONCLUSION

The amount of GAA in nutritional supplements registered in the U.S. National Institutes of Health dietary supplement database varies considerably. GAA‐containing products appear to be a significant source of GAA in human nutrition; further studies are needed to assess the risks and benefits of GAA exposure from nutritional supplements.

## ACKNOWLEDGMENTS

Not applicable.

## FUNDING INFORMATION

None received.

## CONFLICT OF INTEREST

S.M.O. serves as a member of the Scientific Advisory Board on creatine in health and medicine (AlzChem LLC). S.M.O. co‐owns patent “Supplements Based on Liquid Creatine” at European Patent Office (WO2019150323 A1), and has received research support related to creatine and/or guanidinoacetic acid during the past 36 months from the Serbian Ministry of Education, Science, and Technological Development, the Provincial Secretariat for Higher Education and Scientific Research, AlzChem GmbH, ThermoLife International, and Hueston Hennigan LLP. S.M.O. is the founder of Centram, a biotechnology startup developing and commercializing innovative nutraceuticals that can support and rejuvenate energy metabolism, the gut‐brain‐muscle axis, and immunity across various health domains. S.M.O. does not own stocks and shares in any organization. LR, NT and SR declare no conflict of interest.

## ETHICS STATEMENT

Not applicable.

## Data Availability

No new data were described in the manuscript.
